# Biobutanol production from coffee silverskin

**DOI:** 10.1186/s12934-018-1002-z

**Published:** 2018-09-27

**Authors:** María Hijosa-Valsero, Jerson Garita-Cambronero, Ana I. Paniagua-García, Rebeca Díez-Antolínez

**Affiliations:** 1 Centro de Biocombustibles y Bioproductos, Instituto Tecnológico Agrario de Castilla y León (ITACyL), Villarejo de Órbigo, 24358 León, Spain; 2 Instituto de Recursos Naturales (IRENA), Universidad de León, Avenida de Portugal 42, 24071 León, Spain

**Keywords:** Coffee silverskin, Lignocellulosic wastes, Pretreatment, Butanol, ABE fermentation, Biorefinery

## Abstract

**Background:**

Coffee silverskin, a by-product from coffee roasting industries, was evaluated as a feedstock for biobutanol production by acetone–butanol–ethanol fermentation. This lignocellulosic biomass contained approximately 30% total carbohydrates and 30% lignin. Coffee silverskin was subjected to autohydrolysis at 170 °C during 20 min, with a biomass-to-solvent ratio of 20%, and a subsequent enzymatic hydrolysis with commercial enzymes in order to release simple sugars. The fermentability of the hydrolysate was assessed with four solventogenic strains from the genus *Clostridium*. In addition, fermentation conditions were optimised via response surface methodology to improve butanol concentration in the final broth.

**Results:**

The coffee silverskin hydrolysate contained 34.39 ± 2.61 g/L total sugars, which represents a sugar recovery of 34 ± 3%. It was verified that this hydrolysate was fermentable without the need of any detoxification method and that *C. beijerinckii* CECT 508 was the most efficient strain for butanol production, attaining final values of 4.14 ± 0.21 g/L acetone, 7.02 ± 0.27 g/L butanol and 0.25 ± 0.01 g/L ethanol, consuming 76.5 ± 0.8% sugars and reaching a butanol yield of 0.269 ± 0.008 g_B_/g_S_ under optimal conditions.

**Conclusions:**

Coffee silverskin could be an adequate feedstock for butanol production in biorefineries. When working with complex matrices like lignocellulosic biomass, it is essential to select an adequate bacterial strain and to optimize its fermentation conditions (such as pH, temperature or CaCO_3_ concentration).
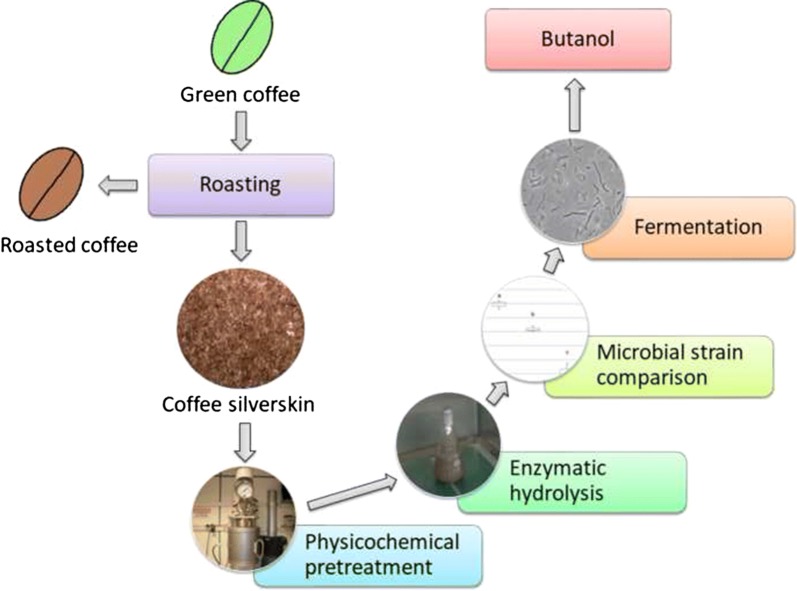

**Electronic supplementary material:**

The online version of this article (10.1186/s12934-018-1002-z) contains supplementary material, which is available to authorized users.

## Background

In a worldwide context of high energetic costs and raw materials scarcity, it is essential to develop industrial processes with low energy requirements and using wastes as feedstocks. In recent years, much attention has been paid to n-butanol production from lignocellulosic wastes by acetone–butanol–ethanol (ABE) fermentation. Butanol has been successfully obtained from a wide range of agricultural and forestry wastes, as well as some energy crops, such as apple pomace, potato peel, brewers’ spent grain, corn cobs, corn stover, corn fiber, Jerusalem artichoke, sweet sorghum bagasse, switchgrass, wheat straw and rice straw, among others [1–8]. This alcohol has numerous applications: solvent, extractant, base-product in chemical industry or fuel [9, 10]. The advantages of butanol as a fuel, in comparison to ethanol, are its higher flash point, higher energy content, lower volatility and lower hygroscopicity; in addition, butanol can be easily mixed with gasoline at any proportion and it can be transported through existing pipelines [9, 10].

Coffee is one of the most consumed beverages worldwide. In 2016, the agricultural area devoted to green coffee cultivation reached 10,975,184 ha, which yielded 9,221,534 t green coffee. The main producing countries were Brazil, Vietnam, Colombia and Indonesia [11]. During the processing of coffee fruits—either by wet or dry methods—the outer skin, pulp, pectic adhesive layer and parchment are removed, whereas the green coffee beans surrounded by an attached silverskin are preserved and sent to roasting industries in consuming countries [12]. Coffee silverskin is a thin tegument obtained as a by-product after the roasting process [13] and it constitutes about 4.2% (w/w) of coffee beans [14]. Coffee silverskin contains important amounts of cellulosic and hemicellulosic fibres [13, 14], as well as interesting molecules such as caffeine and polyphenolic compounds, which makes this by-product an interesting source of cellulose [15], dietary fibres [16] and antioxidants [17, 18], and its use has also been proposed as fuel [19, 20], compost [19, 20], fertilizer [20] and feedstock for amylase or ethanol production by fermentation [13, 21, 22].

In 2016 the European Union (EU-28) imported 2.95 Mt of green coffee and produced about 1.90 Mt of roasted coffee [23]. The coffee silverskin obtained in roasting industries could be an alternative carbohydrate source to be used in butanol biorefineries, due to its continuous production throughout the year, its polysaccharide content and its availability. However, to the best of our knowledge, coffee silverskin has never been successfully employed as a feedstock for ABE fermentation. In the present work, the use of coffee silverskin as a feedstock for biobutanol production was assessed. The aims of this study were to hydrolyze cellulose and hemicellulose from coffee silverskin into simple fermentable sugars and to obtain a broth which could be directly fermentable by solventogenic *Clostridium* strains to produce butanol.

## Methods

### Chemicals and reagents

Analytical grade H_2_SO_4_, HCl and NaOH were obtained from Panreac (Castellar del Vallès, Spain). Citric acid anhydrous was purchased from Sigma-Aldrich (Steinheim, Germany). The enzyme Cellic CTec2, whose enzymatic activity was 124 FPU/mL and protein content was 176 mg/mL, was provided by Novozymes (Tianjin, China).

### Biomass description and processing

Dry coffee silverskin was kindly provided by Illycaffè S.p.A. (Trieste, Italy) in summer 2016. The biomass was ground in a SM100 Comfort rotary mill (Retsch GmbH, Haan, Germany) and sieved to a size of 0.5–1.0 mm.

Moisture, ash, structural carbohydrates (cellulose and hemicellulose), starch, lignin, proteins, fats and total phenolic compounds were analysed as described by Hijosa-Valsero et al. [7]. The chemical composition of coffee silverskin can be found in Table 1.

**Table 1:** Chemical composition of coffee silverskin (dry mass)

Parameter	Content
Total carbohydrates (%)	30.37
Soluble carbohydrates (%)	0.40
Cellulose (%)	10.33
Hemicellulose (%)	9.64
Starch (%)	7.15
Lignin (%)	29.91
Protein (%)	14.43
Fats (%)	4.97
Ash (%)	5.87
Moisture (%)	4.81
Total phenolic compounds (mg/g)	8.0

### Pretreatment of coffee silverskin

#### Physicochemical pretreatment

An autohydrolysis pretreatment was performed with a high-pressure 2-L reactor made of alloy Carpenter-20 (Parr Instrument Company, Moline, IL, USA). Given the total carbohydrate content in coffee silverskin (Table 1), a biomass-to-solvent ratio of 20% (w/w) was chosen to guarantee a sufficient concentration of simple sugars in the final hydrolysate. Therefore, 80 g dry biomass were placed in the reactor container and 320 g distilled water were added. The reaction mixture was heated at a rate of about 7.6 °C/min with continuous stirring, until a temperature of 170 °C was attained. Then, the reactor was kept at that temperature during 20 min. At the end of the process, the reactor was cooled and the solid/liquid mixture was recovered.

#### Enzymatic hydrolysis

After the autohydrolysis, an enzymatic hydrolysis was carried out on the solid/liquid mixture obtained in the reactor. The sample was placed in a 500-mL Erlenmeyer flask, and then 3.88 g citric acid dissolved in 5 mL water were added. The pH was adjusted to 5.0 with a 40% NaOH solution. This made a buffer citrate of about 50 mM and pH 5.0 [24, 25]. Afterwards, 2320 µl of the enzyme Cellic CTec2 (Novozymes, Tianjin, China) were added (approximately 29 µl enzyme per gramme of dry coffee silverskin). The flasks were capped and placed in an orbital shaker at 50 °C and 180 rpm during 72 h. After enzymatic hydrolysis, the samples were filtered and prepared for chemical analyses as explained in section “Chemical analyses of hydrolysates and fermented broths”, in order to quantify simple sugars and potential fermentation inhibitors. This final hydrolysate was subjected to ABE fermentation.

### Fermentation of liquid hydrolysates and strain selection

The strains *Clostridium beijerinckii* CECT 508 (=NCIMB 8052) (CECT, Paterna, Spain), *C. beijerinckii* DSM 6423, *C. saccharobutylicum* DSM 13864 and *C. saccharoperbutylacetonicum* DSM 2152 (DSMZ, Braunschweig, Germany) were assessed for the fermentation of coffee silverskin hydrolysates. Strain culture and inocula preparation for *C. beijerinckii* CECT 508 was performed according to Díez-Antolínez et al. [26]. For the strains *C. beijerinckii* DSM 6423, *C. saccharobutylicum* DSM 13864 and *C. saccharoperbutylacetonicum* DSM 2152, lyophilised cells were resuspended in 10 mL sterile medium containing 19 g/L Reinforced Clostridial Medium—RCM (Oxoid, Basingstoke, UK) and 10 g/L glucose. This medium was incubated during 24 h at 35 °C under anaerobic conditions. Then, 1.5 mL were transferred to a sterile cryogenic vial and 0.4 mL glycerol (80% v/v) were added. The vials were closed, shaken and stored at − 80 °C until being used. For the cellular reactivation of *C. beijerinckii* DSM 6423 and *C. saccharobutylicum* DSM 13864, a loopful of the thawed glycerinate was spread on a Petri dish containing 38 g/L RCM and 20 g/L agar, and the dish was incubated at 35 °C under anaerobic conditions until colonies were visible (1–3 mm). Then, a colony was transferred to 50 mL of sterile liquid medium (19 g/L RCM, 10 g/L glucose). In the case of *C. saccharoperbutylacetonicum* DSM 2152, potato medium [27] was used instead of RCM. Afterwards, gaseous N_2_ was injected into the headspace of the closed bottles during 5 min to obtain anaerobic conditions. The bottles were incubated for 24 h at 35 °C and were employed as inocula, containing an approximate bacterial density of 5·10^8^ cells/mL.

For fermentability tests, coffee silverskin hydrolysates were filtered through a nylon mesh (30 denier) and the filtrate was centrifuged at 2480×*g* during 10 min (centrifuge Jouan CR3i, Château-Gontier, France). Afterwards, hydrolysates were supplemented with 1 or 5 g/L yeast extract, 2.1 g/L NH_4_Cl, 0.5 g/L K_2_HPO_4_, 0.5 g/L KH_2_PO_4_, 0.01 g/L FeSO_4_·7H_2_O, 0.2 g/L MgSO_4_·7H_2_O, 0.5 g/L cysteine and 5 g/L CaCO_3_, autoclaved and adjusted to pH 6.0. Two levels of yeast extract concentration were tested, as this nutrient usually has an important effect on ABE yields. Then, 1.5 mL of the corresponding inoculum were added to 48.5 mL of fermentation medium in rubber-capped bottles, where gaseous N_2_ was bubbled during 5 min to guarantee anaerobic conditions. Fermentation bottles were incubated at 35 °C and 100 rpm in an Infors HT Minitron orbital shaker (Infors AG, Bottmingen, Switzerland) during 96 h. Fermentation controls were prepared with aqueous solutions containing glucose/xylose mixtures at similar concentrations to those of hexoses/pentoses found in coffee silverskin hydrolysates, and supplemented with the abovementioned nutrients. Experiments were performed in triplicate. Bacterial density in fermentation broths was determined with a Bürker counting chamber (Paul Marienfeld GmbH & Co. KG, Lauda-Königshofen, Germany).

### Optimization of fermentation conditions

The best ABE-producing strain according to the procedure described in section “Fermentation of liquid hydrolysates and strain selection” was selected and it was subjected to an experimental design to improve butanol concentrations in the fermentation broth. To this end, a Box-Behnken design linked to the response surface methodology (RSM) was applied to determine the most adequate values for temperature, initial pH and CaCO_3_ concentration during the fermentation (three independent variables) in order to maximize butanol concentration (response variable). The experimental design consisted of 15 experimental runs including three central points. Coffee silverskin hydrolysate (supplemented with 1 g/L yeast extract and the nutrients described in section “Fermentation of liquid hydrolysates and strain selection”) was fermented under the fifteen different conditions during 96 h, and the final butanol concentrations were measured to be employed as input for the RSM model. A response surface was calculated and the resulting equation was used to estimate the optimal temperature, initial pH and CaCO_3_ concentration values to obtain the highest amount of butanol. Afterwards, the mathematically estimated optimal points were validated by performing fermentation experiments in triplicate. More details on the experimental design can be found in Additional file 1.

### Chemical analyses of hydrolysates and fermented broths

Aqueous samples of hydrolysates and fermented broths were centrifuged, filtered and analyzed according to Hijosa-Valsero et al. [7] for the quantification of sugars (cellobiose, glucose, xylose, rhamnose and arabinose), potential fermentation inhibitors (formic acid, acetic acid, levulinic acid, 5-hydroxymethylfurfural (5-HMF), furfural and total phenolic compounds) and ABE fermentation products (acetone, butanol, ethanol, acetic acid and butyric acid). Fermentation yields (Y_i/S_, g/g) were expressed as the ratio between the metabolite (i) produced and the total sugars consumed (S). Metabolite productivity rates (W_i_, g/(L·h)) were calculated as the ratio between the metabolite (i) expressed in concentration (g/L) and the fermentation time (h). Sugar recovery or sugar conversion efficiency (%) was obtained as the ratio between the mass of simple sugars in the hydrolysate and the total mass of carbohydrates in the untreated coffee silverskin (the volume of the hydrolysate was measured after filtration in order to make this calculation).

### Statistical analyses

Comparisons among samples were assessed with a one-way ANOVA and Tukey’s HSD test using the software Statistica 7 (StatSoft Inc., Tulsa, OK, USA). Box-Behnken RSM experimental designs were performed with the software Minitab 16 (Minitab Inc., State College, PA, USA).

## Results and discussion

### Coffee silverskin hydrolysis

The initial total carbohydrate content of the studied coffee silverskin was about 30% (Table 1), which is lower than values described by other authors, who reported cellulose contents of 18–24% [13, 14], hemicellulose contents of 13–17% [13, 14] or total fibre contents of 54–62% [12–14, 16]. Because of this, a biomass-to-solvent ratio of 20% was used during the pretreatment, in order to guarantee a sufficient amount of total carbohydrates in the hydrolysate. Table 2 shows the chemical composition of coffee silverskin hydrolysates after the autohydrolysis and after the subsequent enzymatic hydrolysis. Autohydrolysis alone only released 6.54 g/L simple sugars, but it contributed to cellulose and hemicellulose degradation, which were partially hydrolyzed during the enzymatic treatment, thus producing a final hydrolysate with 34.39 g/L total sugars. Sugar recovery efficiency was relatively low (33.74 ± 3.49%), but it can be considered successful in comparison to previous data. For instance, Niglio et al. [28] treated coffee silverskin with a combination of H_2_SO_4_ and ultrasounds, followed by enzymatic hydrolysis, and attained an approximate sugar recovery yield of 18% working with 3% biomass, and 16% sugar yield working with 10% biomass. Taking into account that the present work was performed with an initial biomass content of 20%, and that sugar recovery yields are inversely related to biomass amounts, then autohydrolysis can be proposed as an appropriate pretreatment for coffee silverskin. Caffeine concentrations in the hydrolysates (Table 2) are consistent with reported caffeine contents in coffee silverskin [17].

**Table 2:** Composition of coffee silverskin hydrolysates after each pretreatment step, expressed in g/L

	Autohydrolysis	Autohydrolysis + enzymatic hydrolysis
Cellobiose	0.56 ± 0.25	0.62 ± 0.07
Glucose	0.51 ± 0.10	21.83 ± 0.81
Xylose	1.73 ± 0.28	9.44 ± 1.71
Rhamnose	2.65 ± 0.33	1.01 ± 1.00
Arabinose	1.11 ± 0.25	1.49 ± 0.56
Total sugars	6.54 ± 0.80	34.39 ± 2.61
Formic acid	1.76 ± 0.26	1.64 ± 0.20
Acetic acid	1.32 ± 0.14	1.94 ± 0.05
Levulinic acid	0.00 ± 0.00	0.00 ± 0.00
5-HMF	0.09 ± 0.02	0.07 ± 0.01
Furfural	0.08 ± 0.05	0.09 ± 0.01
Phenolic compounds	1.51 ± 0.00	1.52 ± 0.03
Caffeine	0.91 ± 0.07	0.82 ± 0.04

Means and standard deviations are provided. Autohydrolysis conditions: 170 °C, 20 min, 20% solid biomass. Xylose concentration is in fact a sum of xylose, galactose and mannose concentrations

The combination of autohydrolysis and enzymatic hydrolysis for lignocellulosic biomass degradation and subsequent fermentation has been explored by different authors. Buruiana et al. [29] pretreated corn stover (10% p/v) with autohydrolysis at 180–223 °C, followed by a treatment with β-glucosidase, obtaining a yield of 12.6% soluble sugars. The resulting broth was fermented by *Saccharomyces cerevisiae* CECT 1170, producing 51.6 g/L ethanol. Amiri and Karimi [24] subjected pine and elm wood (10% w/v) to autohydrolysis at 180 °C during 60 min, and then to an enzymatic hydrolysis with Celluclast 1.5 L (cellulase) and Novozym 188 (β-glucosidase), releasing about 20% soluble sugars. Pine wood hydrolysate, with an initial sugar concentration of 20.8 g/L, was fermented by *C. acetobutylicum* NRRL B-591 and yielded 3.5 g/L butanol, 2 g/L acetone and 0.4 g/L ethanol, whereas elm wood hydrolysate (23.2 g/L initial sugars) only produced 2.5 g/L ABE. Gonçalves et al. [25] performed an autohydrolysis (200 °C, 50 min) on green coconut shells, followed by an enzymatic treatment with Cellic CTec2 and Cellic HTec2, resulting in a sugar release of 91% from cellulose. The hydrolysates were fermented by *Pichia stipitis* Y7124, *S. cerevisiae* PE2 or *Zymomonas mobilis* B14023, obtaining ethanol concentrations of 7.30–8.78 g/L [yields 0.43–0.45 g/g; productivity 0.15–0.18 g/(L·h)].

### Coffee silverskin fermentation

The four strains (CECT 508, DSM 6423, DSM 13864 and DSM 2152) were able to grow in coffee silverskin hydrolysates, as denoted by their total sugar consumption above 50% (Table 3), although there were important differences in ABE fermentation performance depending on the strain (Table 3). *Clostridium beijerinckii* CECT 508 was the most efficient strain, followed by *C. beijerinckii* DSM 6423, *C. saccharoperbutylacetonicum* DSM 2152 and *C. saccharobutylicum* DSM 13864 (Table 3). Actually, *C. beijerinckii* CECT 508 was statistically superior (p < 0.05) to the other strains in terms of ABE production under 1 g/L yeast extract, obtaining 3.28 g/L acetone, 5.94 g/L butanol and 0.28 g/L ethanol, with a butanol yield of 0.245 g/g and a butanol productivity of 0.062 g/(L·h) (Table 3), and butanol production from coffee silverskin hydrolysates by *C. beijerinckii* CECT 508 under 5 g/L yeast extract was statistically higher (p < 0.07) than that of the strains *C. saccharobutylicum* DSM 13864 and *C. saccharoperbutylacetonicum* DSM 2152. Regarding the role of yeast extract concentration on ABE performance, no significant differences (p < 0.05) were observed between coffee silverskin hydrolysates containing 1 or 5 g/L yeast extract, for any of the four strains (Table 3). Therefore, in order to reduce costs, it is preferable to add only 1 g/L yeast extract.

**Table 3:** Fermentation results for coffee silverskin hydrolysates with four different bacterial strains at two levels of yeast extract concentration

Strain	Yeast extract (g/L)	Sample	Bacterial growth	ABE fermentation metabolites						Sugar consumption	Yield and productivity	
			Density (cell/mL)	Acetone (g/L)	Butanol (g/L)	Ethanol (g/L)	Acetic acid (g/L)	Butyric acid (g/L)	Isopropanol (g/L)	Total sugars (%)	Y _B/S_ (g/g)	W_B_ (g/L·h)
CECT 508	1	Control	3.5·10^8^	2.15 ± 0.66	6.55 ± 0.43	0.10 ± 0.02	0.61 ± 0.03	0.62 ± 0.20	0 ± 0	93 ± 6	0.219 ± 0.029	0.068 ± 0.004
	1	Coffee silverskin	2.5·10^8^	3.28 ± 0.29	5.94 ± 0.29	0.28 ± 0.01	1.96 ± 0.23	0.35 ± 0.04	0 ± 0	79 ± 3	0.245 ± 0.004	0.062 ± 0.003
	5	Control	6.7·10^8^	1.05 ± 0.13	6.52 ± 0.10	0.05 ± 0.00	1.10 ± 0.03	1.00 ± 0.44	0 ± 0	94 ± 1	0.202 ± 0.001	0.068 ± 0.001
	5	Coffee silverskin	3.8·10^8^	1.62 ± 0.10	5.60 ± 0.23	0.13 ± 0.01	3.31 ± 0.04	1.86 ± 0.26	0 ± 0	79 ± 5	0.207 ± 0.010	0.058 ± 0.002
DSM 6423	1	Control	1.1·10^9^	0 ± 0	5.98 ± 0.14	0.08 ± 0.00	0.74 ± 0.06	0.52 ± 0.13	1.68 ± 0.04	94 ± 3	0.185 ± 0.011	0.062 ± 0.001
	1	Coffee silverskin	4.1·10^8^	0 ± 0	4.18 ± 0.20	0.11 ± 0.00	3.87 ± 0.29	1.53 ± 0.19	1.93 ± 0.06	66 ± 2	0.184 ± 0.004	0.044 ± 0.002
	5	Control	9.8·10^8^	0.03 ± 0.04	6.77 ± 0.46	0.11 ± 0.01	0.74 ± 0.08	0.46 ± 0.14	2.46 ± 0.21	100 ± 0	0.198 ± 0.014	0.070 ± 0.005
	5	Coffee silverskin	3.5·10^8^	0 ± 0	3.53 ± 0.89	0.08 ± 0.01	n.q.	2.20 ± 0.11	0.79 ± 0.12	62 ± 1	0.166 ± 0.040	0.037 ± 0.009
DSM 13864	1	Control	8.0·10^8^	2.36 ± 0.10	6.52 ± 0.34	0.21 ± 0.01	0.54 ± 0.12	0.18 ± 0.00	0 ± 0	99 ± 0	0.191 ± 0.010	0.068 ± 0.004
	1	Coffee silverskin	4.3·10^8^	0.75 ± 0.28	1.13 ± 0.53	0.08 ± 0.02	n.q.	2.53 ± 1.14	0 ± 0	54 ± 5	0.060 ± 0.024	0.012 ± 0.006
	5	Control	7.1·10^8^	1.63 ± 0.04	7.13 ± 0.24	0.13 ± 0.00	0.60 ± 0.09	0.58 ± 0.13	0 ± 0	100 ± 0	0.209 ± 0.007	0.074 ± 0.003
	5	Coffee silverskin	5.0·10^8^	1.71 ± 0.99	3.00 ± 1.80	0.13 ± 0.04	n.q.	2.27 ± 1.06	0 ± 0	75 ± 10	0.111 ± 0.060	0.031 ± 0.019
DSM 2152	1	Control	5.9·10^8^	2.53 ± 0.06	6.63 ± 0.07	0.25 ± 0.03	0.64 ± 0.01	0.26 ± 0.01	0 ± 0	97 ± 0	0.200 ± 0.002	0.069 ± 0.001
	1	Coffee silverskin	2.1·10^8^	1.94 ± 0.13	2.43 ± 0.09	0.10 ± 0.01	n.q.	1.88 ± 0.10	0 ± 0	58 ± 2	0.123 ± 0.008	0.025 ± 0.001
	5	Control	1.1·10^9^	1.87 ± 0.02	7.38 ± 0.32	0.47 ± 0.17	0.48 ± 0.01	0.25 ± 0.00	0 ± 0	94 ± 3	0.228 ± 0.004	0.077 ± 0.003
	5	Coffee silverskin	2.3·10^8^	1.59 ± 0.26	2.87 ± 0.54	0.13 ± 0.02	n.q.	0.85 ± 0.18	0 ± 0	52 ± 7	0.160 ± 0.011	0.030 ± 0.006

Controls consist of aqueous solutions containing sugars and nutrients at similar concentrations to those of coffee silverskin hydrolysates. n.q. Not quantified due to HPLC coelution interferences in that sample

The toxic effect of coffee silverskin hydrolysates on the four strains tested was visible on sugar utilization, since total sugar consumption was 93–100% for control samples and 52–79% for hydrolysates (Table 3). In addition, an evident negative effect on cell growth was observed for bacteria developing in coffee silverskin hydrolysate in comparison to their respective controls (Table 3). Cell density was 1.40–1.86 times higher in control samples than in hydrolysates for the strains *C. beijerinckii* CECT 508 and *C. saccharobutylicum* DSM 13864, and 2.68–4.78 times higher for *C. beijerinckii* DSM 6423 and *C. saccharoperbutylacetonicum* DSM 2152. The strains *C. beijerinckii* DSM 6423, *C. saccharobutylicum* DSM 13864 and *C. saccharoperbutylacetonicum* DSM 2152 suffered a significant decrease in butanol production when comparing coffee silverskin hydrolysates and control fermentations, regardless of their initial yeast extract concentration (p < 0.05). On the contrary, the strain *C. beijerinckii* CECT 508 produced 5.94 g/L butanol from coffee silverskin hydrolysates under 1 g/L yeast extract, a value which was not significantly different (p < 0.05) from the concentration of 6.55 g/L obtained in its control sample (Table 3). This could indicate that this particular strain is more tolerant to the possible inhibitors found in the hydrolysates (Table 2). It was experimentally determined that *C. beijerinckii* CECT 508 did not consume caffeine during the fermentation of these hydrolysates (caffeine concentration in the broth after 96 h of fermentation was 0.85 ± 0.01 g/L). Some bacteria have evolved to metabolize caffeine, due to the frequent presence of this plant-origin compound in the environment [30]. On the other hand, several studies report growth inhibition or metabolic interference in certain bacterial groups caused by caffeine [31, 32], as well as metabolic changes such as the stimulation of sporulation or the inhibition of macromolecular synthesis in other *Clostridium* species [33, 34]. Moreover, the concentration of phenolic compounds did not vary during the fermentation (final concentration 1.51 ± 0.01 g/L), thus indicating that *C. beijerinckii* CECT 508 did not metabolize this type of inhibitory substances. Therefore, further research in this matter, regarding solventogenic Clostridia, could be useful in order to improve butanol generation in the three less productive strains found in the present work.

### Optimization of fermentation conditions with the selected strain

**Fig. 1:**
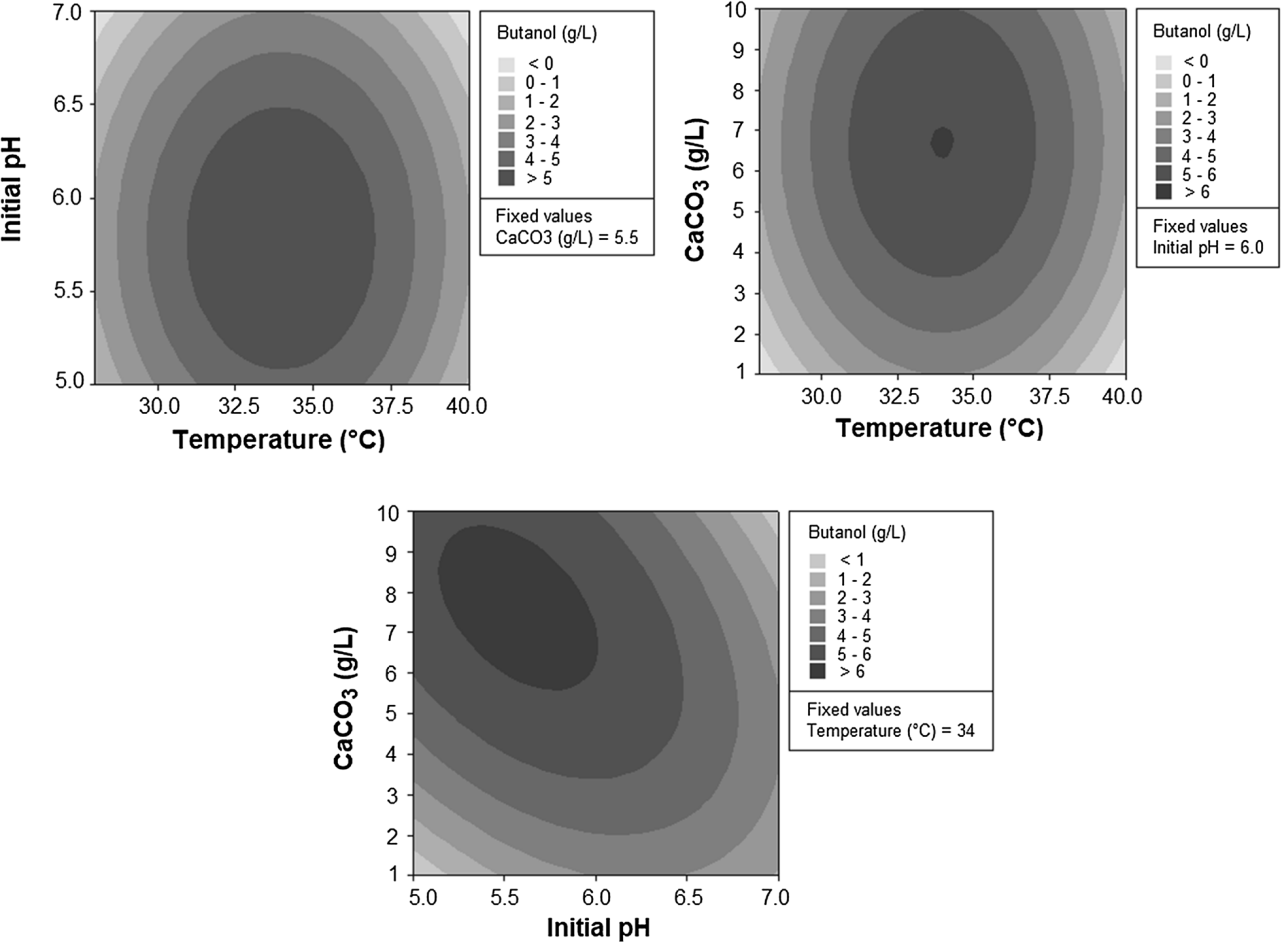
Contour plots for the estimation of butanol concentration as a function of fermentation conditions (temperature, initial pH and CaCO_3_ concentration), according to the mathematical RSM model

In spite of the relatively low initial sugar concentration (34 g/L), coffee silverskin hydrolysate was successfully fermented into 5.94 ± 0.29 g/L butanol by one of the strains (*C. beijerinckii* CECT 508). In order to check the possibility of improving this butanol concentration when fermenting coffee silverskin hydrolysates with *C. beijerinckii* CECT 508, the most appropriate values for temperature, initial pH and CaCO_3_ concentration during fermentation were optimized via RSM. The fifteen fermentation experiments produced variable butanol amounts, ranging from 0 to 7.42 g/L (Additional file 1: Table S2). These data were used to build a mathematical model (Fig. 1) which explained 88.63% of the variation, but its predicted R-square value of 0% indicated that the model was overfit (Additional file 1: Table S6).

The equation of the model (Additional file 1: Table S6) was used to calculate the optimal values of temperature, initial pH and CaCO_3_ concentration which would yield the highest butanol concentration. The optimization output indicated that the best fermentation parameters would be 33.9 °C, initial pH 5.59 and 7.55 g/L CaCO_3_, and the estimated butanol concentration under those conditions would be 6.30 g/L.

The model was validated experimentally by fermenting coffee hydrolysates under the optimal conditions (33.9 °C, pH 5.59 and 7.55 g/L CaCO_3_) in triplicate. This validation gave a result of 7.02 ± 0.27 g/L butanol (Table 4), which is significantly higher (p < 0.05) than the previously recorded value of 5.94 ± 0.29 g/L butanol before optimization (section “Coffee silverskin fermentation”). In spite of the real improvement obtained, it must be noted that the RSM model was not very accurate to estimate butanol production (6.30 g/L estimated vs. 7.02 g/L experimental). This could be due to the abovementioned overfitting. The reasons underlying this fact may be related to the variable composition of coffee silverskin hydrolysates depending on the batch (Table 2) or to the changeable responses of bacterial spore batches used to prepare the inocula on different days.

**Table 4:** Fermentation of coffee silverskin hydrolysates with *C. beijerinckii* CECT 508 under optimal conditions (33.9 °C, pH 5.59 and 7.55 g/L CaCO_3_) after 96 h

ABE fermentation metabolites	
Acetone (g/L)	4.14 ± 0.21
Butanol (g/L)	7.02 ± 0.27
Ethanol (g/L)	0.25 ± 0.01
Acetic acid (g/L)	2.42 ± 0.05
Butyric acid (g/L)	0.57 ± 0.14
Sugar consumption	
Total sugars (%)	76.5 ± 0.8
Yield and productivity	
Y _B/S_ (g/g)	0.269 ± 0.008
W_B_ [g/(L·h)]	0.073 ± 0.003

Regarding the calculated values for the maximum butanol production (33.9 °C, initial pH 5.59 and 7.55 g/L CaCO_3_), all of them are within the normal ranges for conventional ABE fermentations. Optimal fermentation temperatures depend on the nature of the feedstock, and they can lie between 29 and 39 °C [35]. Good levels of solvent production have been obtained for pH ranges of 5.0–6.5 [35], but pH-dependence varies from strain to strain [36]. When CaCO_3_ is used in fermentation broths for pH control, its concentration is about 5 g/L [26, 37]. In a recent review, Ujor et al. [38] discussed that CaCO_3_ does not only work as a buffer during ABE fermentations, but it could also favor the concomitant use of glucose and xylose, enhance butanol tolerance and reduce the toxic effects of lignocellulosic biomass hydrolysates.

To the best of our knowledge, coffee by-products had never been transformed into biobutanol yielding such high titers. The concentration obtained under optimal conditions with coffee silverskin hydrolysates (7.02 g/L butanol) can be considered in the medium–high range of the values that are usually obtained from lignocellulosic biomass [39–42]. For instance, other agricultural or food wastes have been proven to be suitable feedstocks for the production of this solvent, like corn stover (8.98 g/L butanol) [43], apple pomace (9.11 g/L butanol) [7], grape marc (6 g/L butanol) [44], or wheat straw (12.0 g/L butanol) [45]. In addition, there is still room for a further optimization of the global process to obtain butanol from coffee silverskin. For instance, the adjustment of enzymatic hydrolysis and the concentration of its buffering solution is known to have an important effect on butanol generation [46, 47].

The possibility of directly fermenting coffee silverskin without the need of expensive detoxification steps makes this biomass attractive for biorefineries. In addition, coffee silverskin may be mixed with other food or agricultural wastes with a higher carbohydrate composition, thus enabling the production of hydrolysates richer in sugar contents, which could lead to higher butanol concentrations in the fermentation broth. This would significantly reduce butanol separation and purification costs at biorefineries with conventional or alternative methods such as gas stripping, pervaporation, adsorption, liquid–liquid extraction or combined techniques [48]. For instance, in the case of gas stripping it is advisable that butanol is present in the broth at concentrations higher than 8 g/L to save energy [46, 49, 50].

## Conclusions

Coffee silverskin could be an adequate feedstock for ABE fermentation in biorefineries. Its physicochemical pretreatment by autohydrolysis is technically easy and environmentally friendly, because it does not require any reagent but water. Another advantage of the developed treatment is the absence of detoxification steps, which simplifies the process and makes it more economically feasible. However, further research is still needed to implement butanol production from lignocellulosic wastes at industrial scale, especially in order to achieve higher butanol concentrations during ABE fermentation and to improve butanol separation and purification technologies.

## Additional file

**Additional file 1.** Details on statistical processes for optimizing the fermentation are supplied as additional information.

## Abbreviations

ABE: acetone–butanol–ethanol
RSM: response surface methodology
