# Supplementary material for: Biobutanol production from coffee silverskin
Source: Microb Cell Fact. 2018 Sep 27;17:154. doi: 10.1186/s12934-018-1002-z (PMC6158808; doi:10.1186/s12934-018-1002-z)
Supplement: Supplementary file 1 — Additional file 1. Details on statistical processes for optimizing the fermentation are supplied as additional information. [file 12934_2018_1002_MOESM1_ESM.docx]

**ADDITIONAL INFORMATION**

**Biobutanol production from coffee silverskin**

María Hijosa-Valsero^1^, Jerson Garita-Cambronero^1^, Ana I. Paniagua-García^1,2^, Rebeca Díez-Antolínez^1,2^

^1^ Centro de Biocombustibles y Bioproductos, Instituto Tecnológico Agrario de Castilla y León (ITACyL), Villarejo de Órbigo, 24358 León, Spain

^2^ Instituto de Recursos Naturales (IRENA), Universidad de León, Avenida de Portugal 42, 24071 León, Spain

**Optimization of fermentation conditions**

A Box-Behnken design linked to the Response Surface Methodology (RSM) was applied to determine the most adequate values for temperature, initial pH and CaCO_3_ concentration during the fermentation (three independent variables) in order to maximize butanol concentration (response variable). The experimental design consisted of 15 experimental runs, including 3 central points, and had 3 factors (independent variables), 1 replicate and 1 block. The value ranges for each independent variable are shown in Table S1. The different conditions tested and their corresponding experimental responses are provided in Table S2.

The fifteen fermentation experiments produced variable butanol amounts, ranging from 0 to 7.42 g/L (Table S2). The central replicate points (runs 2, 7 and 15 in Table S2) generated butanol concentrations of 5.83-6.34 g/L. The statistical analyses of the data showed that two of the terms (T*pH and T*CaCO_3_) were not statistically significant due to their high p values (Tables S3, S4 and S5). Therefore, those two terms were removed and the model was recalculated (Tables S6, S7 and S8). The new model explained 88.63% of the variation, but its predicted R-square value of 0% indicated that the model was overfit (Table S6). Another relevant observation was the fact that runs 6 and 12 (Table S2) were considered outliers by the model (Table S8), which implies that their weight in the model equation will be negligible. Because of that, the highest butanol recorded value (7.42 g/L, run 6) is not represented in the model or in the contour plots (Figure 1).

Hence, when the equation was used to calculate the optimal values of temperature, initial pH and CaCO_3_ concentration which would yield the highest butanol concentration, the model considered that the optimal conditions would lie near the central point. Specifically, the optimization output indicated that the optimal fermentation parameters would be 33.9°C, initial pH 5.59 and 7.55 g/L CaCO_3_, and the estimated butanol concentration under those conditions would be 6.30 g/L.

The model was validated experimentally by fermenting coffee hydrolysates under the optimal conditions (33.9°C, pH 5.59 and 7.55 g/L CaCO_3_). However, taking into account the abovementioned considerations about the outliers and their possible underestimation in the model, it was decided to verify whether the result of run 6 (Table S2) was indeed an outlier or on the contrary it was an adequate and repeatable fermentation condition. In both cases, the validation was performed in triplicate. The validation of run 6 produced 4.36 ± 1.43 g/L butanol, which clearly indicates that these fermenting conditions were not very repeatable.

Table S1. Minimum and maximum ranges for the independent variables in the RSM experimental design.

|  | Minimum | Maximum |
| --- | --- | --- |
| Temperature (°C) | 28 | 40 |
| Initial pH | 5 | 7 |
| CaCO_3_ (g/L) | 1 | 10 |

Table S2. Fermentation experiments performed in the Box-Behnken RSM design and butanol concentrations observed for each condition. (^a^) Null values were below the quantification limit of 0.05 g/L.

|  | Independent variables (factors) | | | Response |
| --- | --- | --- | --- | --- |
| Run | Temperature (°C) | Initial pH | CaCO_3_ (g/L) | Butanol (g/L) |
| 1 | 34 | 7 | 10 | 0.41 |
| 2 | 34 | 6 | 5.5 | 5.83 |
| 3 | 40 | 6 | 10 | 0.06 |
| 4 | 28 | 7 | 5.5 | 0 ^a^ |
| 5 | 40 | 7 | 5.5 | 0 ^a^ |
| 6 | 34 | 5 | 10 | 7.42 |
| 7 | 34 | 6 | 5.5 | 6.34 |
| 8 | 40 | 5 | 5.5 | 0 ^a^ |
| 9 | 28 | 5 | 5.5 | 0 ^a^ |
| 10 | 40 | 6 | 1 | 0 ^a^ |
| 11 | 34 | 5 | 1 | 0.07 |
| 12 | 34 | 7 | 1 | 0.15 |
| 13 | 28 | 6 | 1 | 0.05 |
| 14 | 28 | 6 | 10 | 0.38 |
| 15 | 34 | 6 | 5.5 | 5.47 |

Table S3. Estimated regression coefficients of Butanol (g/L). *Note*: This model includes all the terms.

| Term | Coefficient | Standard error coef. | T | p |
| --- | --- | --- | --- | --- |
| Constant | -202.717 | 50.4100 | -4.021 | 0.010 |
| Temperature (°C) | 7.344 | 1.7828 | 4.120 | 0.009 |
| Initial pH | 25.240 | 11.0812 | 2.278 | 0.072 |
| CaCO_3_ (g/L) | 3.688 | 1.5476 | 2.383 | 0.063 |
| Temperature (°C) * Temperature (°C) | -0.108 | 0.0232 | -4.644 | 0.006 |
| Initial pH * Initial pH | -1.995 | 0.8365 | -2.385 | 0.063 |
| CaCO_3_ (g/L) * CaCO_3_ (g/L) | -0.092 | 0.0413 | -2.238 | 0.075 |
| Temperature (°C) * Initial pH | -0.000 | 0.1339 | -0.000 | 1.000 |
| Temperature (°C) * CaCO_3_ (g/L) | -0.002 | 0.0298 | -0.084 | 0.936 |
| Initial pH * CaCO_3_ (g/L) | -0.394 | 0.1786 | -2.205 | 0.079 |
|  |  |  |  |  |
| S = 1.60739 | PRESS = 201.440 |  |  |  |
| R-square = 88.65% | R-square(pred.) = 0.00% | R-square(adjusted) = 68.22% | |  |

Table S4. Analysis of variance of Butanol (g/L). *Note*: This model includes all the terms.

| Source | df | Sum of squares Seq. | Sum of squares Adjust. | Mean squares Adjust. | F | p |
| --- | --- | --- | --- | --- | --- | --- |
| Regression | 9 | 100.896 | 100.896 | 11.2107 | 4.34 | 0.060 |
| Lineal | 3 | 14.020 | 58.348 | 19.4493 | 7.53 | 0.027 |
| Temperature (°C) | 1 | 0.017 | 43.848 | 43.8481 | 16.97 | 0.009 |
| Initial pH | 1 | 6.003 | 13.404 | 13.4045 | 5.19 | 0.072 |
| CaCO_3_ (g/L) | 1 | 8.000 | 14.671 | 14.6707 | 5.68 | 0.063 |
| Quadratic | 3 | 74.291 | 74.291 | 24.7637 | 9.58 | 0.016 |
| Temperature (°C) * Temperature (°C) | 1 | 48.619 | 55.729 | 55.7288 | 21.57 | 0.006 |
| Initial pH * Initial pH | 1 | 12.725 | 14.695 | 14.6955 | 5.69 | 0.063 |
| CaCO_3_ (g/L) * CaCO_3_ (g/L) | 1 | 12.946 | 12.946 | 12.9462 | 5.01 | 0.075 |
| Interaction | 3 | 12.585 | 12.585 | 4.1951 | 1.62 | 0.296 |
| Temperature (°C) * Initial pH | 1 | 0.000 | 0.000 | 0.0000 | 0.00 | 1.000 |
| Temperature (°C) * CaCO_3_ (g/L) | 1 | 0.018 | 0.018 | 0.0182 | 0.01 | 0.936 |
| Initial pH * CaCO_3_ (g/L) | 1 | 12.567 | 12.567 | 12.5670 | 4.86 | 0.079 |
| Residual error | 5 | 12.918 | 12.918 | 2.5837 |  |  |
| Lack of fit | 3 | 12.536 | 12.536 | 4.1788 | 21.87 | 0.044 |
| Pure error | 2 | 0.382 | 0.382 | 0.1911 |  |  |
| Total | 14 | 113.815 |  |  |  |  |

Table S5. Unusual observations of Butanol (g/l). *Note*: This model includes all the terms.

| Obs | Butanol (g/l) | Fit | SE Fit | Resid. | Std. Resid. |  |
| --- | --- | --- | --- | --- | --- | --- |
| 6 | 7.42 | 5.651 | 1.392 | 1.769 | 2.20 | R |
| 12 | 0.15 | 1.919 | 1.392 | -1.769 | -2.20 | R |

R denotes an observation with a large standardized residue.

Table S6. Estimated regression coefficients of Butanol (g/l), after removing the terms with high p values.

| Term | Coefficient | Standard error coef. | T | p |
| --- | --- | --- | --- | --- |
| Constant | -202.250 | 35.5165 | -5.695 | 0.001 |
| Temperature (°C) | 7.331 | 1.3387 | 5.476 | 0.001 |
| Initial pH | 25.240 | 8.5438 | 2.954 | 0.021 |
| CaCO_3_ (g/L) | 3.603 | 0.9902 | 3.638 | 0.008 |
| Temperature (°C) * Temperature (°C) | -0.108 | 0.0197 | -5.491 | 0.001 |
| Initial pH * Initial pH | -1.995 | 0.7075 | -2.820 | 0.026 |
| CaCO_3_ (g/L) * CaCO_3_ (g/L) | -0.092 | 0.0349 | -2.647 | 0.033 |
| Initial pH * CaCO_3_ (g/L) | -0.394 | 0.1510 | -2.608 | 0.035 |
|  |  |  |  |  |
| S = 1.35945 | PRESS = 126.193 |  |  |  |
| R-square = 88.63% | R-square(pred.) = 0.00% | R-square(adjusted) = 77.27% | |  |

Table S7. Analysis of variance of Butanol (g/l), after removing the terms with high p values.

| Source | df | Sum of squares Seq. | Sum of squares Adjust. | Mean squares Adjust. | F | p |
| --- | --- | --- | --- | --- | --- | --- |
| Regression | 7 | 100.878 | 100.878 | 14.4112 | 7.80 | 0.007 |
| Lineal | 3 | 14.020 | 86.315 | 28.7717 | 15.57 | 0.002 |
| Temperature (°C) | 1 | 0.017 | 55.413 | 55.4127 | 29.98 | 0.001 |
| Initial pH | 1 | 6.003 | 16.129 | 16.1288 | 8.73 | 0.021 |
| CaCO_3_ (g/L) | 1 | 8.000 | 24.465 | 24.4651 | 13.24 | 0.008 |
| Quadratic | 3 | 74.291 | 74.291 | 24.7637 | 13.40 | 0.003 |
| Temperature (°C) * Temperature (°C) | 1 | 48.619 | 55.729 | 55.7288 | 30.15 | 0.001 |
| Initial pH * Initial pH | 1 | 12.725 | 14.695 | 14.6955 | 7.95 | 0.026 |
| CaCO_3_ (g/L) * CaCO_3_ (g/L) | 1 | 12.946 | 12.946 | 12.9462 | 7.01 | 0.033 |
| Interaction | 1 | 12.567 | 12.567 | 12.5670 | 6.80 | 0.035 |
| Initial pH * CaCO_3_ (g/L) | 1 | 12.567 | 12.567 | 12.5670 | 6.80 | 0.035 |
| Residual error | 7 | 12.937 | 12.937 | 1.8481 |  |  |
| Lack of fit | 5 | 12.555 | 12.555 | 2.5109 | 13.14 | 0.072 |
| Pure error | 2 | 0.382 | 0.382 | 0.1911 |  |  |
| Total | 14 | 113.815 |  |  |  |  |

Table S8. Unusual observations of Butanol (g/l), after removing the terms with high p values.

| Obs | Butanol (g/l) | Fit | SE Fit | Resid. | Std. Resid. |  |
| --- | --- | --- | --- | --- | --- | --- |
| 6 | 7.42 | 5.651 | 1.177 | 1.769 | 2.60 | R |
| 12 | 0.15 | 1.919 | 1.177 | -1.769 | -2.60 | R |

R denotes an observation with a large standardized residue.
